# Is Long-Term Benzodiazepine Use a Risk Factor for Cognitive Decline? Results of a Systematic Review

**DOI:** 10.1155/2020/1569456

**Published:** 2020-01-23

**Authors:** Danilo Nader, Linda Gowing

**Affiliations:** ^1^University of Adelaide, Adelaide, SA 5005, Australia; ^2^Discipline of Pharmacology, University of Adelaide, Adelaide, SA 5005, Australia

## Abstract

**Background and Aims:**

Benzodiazepines have been widely used for long periods of time despite their adverse effects. The acute effects on cognition are well established. However, less is known about the long-term effects. This study critically reviewed existing evidence of the association between long-term exposure to benzodiazepines and risk of cognitive decline in adults.

**Methods:**

A systematic review with narrative synthesis was conducted. PubMed and PsycINFO databases were searched using combinations of keywords related to “benzodiazepines” and “cognitive function” from database inception to 12 February 2018 to identify prospective longitudinal studies. The records were evaluated for relevance according to the inclusion and exclusion criteria.

**Results:**

Fourteen studies involving 2145 long-term benzodiazepine users were included. Meta-analysis was not undertaken because the combined result would not be meaningful as the included studies differed in several key aspects such as frequency and duration of benzodiazepine use, follow-up periods, cognitive domains, cognitive tests, scoring systems, and statistical analysis. The definition of long-term benzodiazepine use was problematic in all the studies. The exposure was determined by measures which were assumed to represent the whole period in-between the follow-ups. Only 3 of the 14 studies provided support for an association between long-term benzodiazepine use and cognitive decline with a small to medium effect size. However, these three studies used different methods to assess the strength of this association. Global cognitive functioning, verbal memory, intelligence, psychomotor speed, and speed of processing were the cognitive domains affected which also varied across these three studies.

**Conclusions:**

Little evidence of an association between long-term benzodiazepine use and a higher risk of cognitive decline among the general adult population was found. However, discrepancies among the results and inconsistencies regarding the cognitive domains affected and methodological limitations prevent definite conclusions. Therefore, future research with prospective studies specially designed would be of great value.

## 1. Introduction

The prevalence of long-term use of benzodiazepine (BZD) worldwide, including the BZD related drugs called Z-drugs, is approximately 3% in the general population despite the repeated warnings over their safety and lack of evidence of long-term effectiveness [1–3]. The acute cognitive effects of BZDs are well established [3, 4]. However, the long-term impact on cognition is still an area of debate, despite epidemiological evidence which suggests that exposure to BZD might be a risk factor for cognitive impairment and cognitive decline [3, 5, 6]. Cognitive impairment refers to impairments in one or more cognitive functions according to a cognitive performance cutoff point and measured at a single time point whereas cognitive decline involves any deterioration in cognitive function over time according to change in cognitive performance between baseline and follow-up [7, 8]. Cognitive decline is more difficult to assess properly than cognitive impairment as its definition is sometimes arbitrary depending on the cognitive test and the statistical approach used [7]. For this reason, cognitive decline associated with long-term BZD use has been assessed by few prospective longitudinal studies.

Verdoux et al. [9] explored the association between long-term BZD use and cognitive decline through a literature review of prospective studies with unselected subjects from general population and concluded that there were many inconsistencies across the studies. Methodological issues such as differences in sampling method, nonspecification of parameters of BZD use, variation in the definition of BZD long-term exposure, cognitive assessment, follow-up characteristics, potential confounders, and statistical analyses might have compromised the comparability of findings [9, 10].

Also, a series of meta-analyses of the effects of long-term BZD use (≥1 year) on cognition were conducted by Barker et al. in 2004 [11, 12] and updated by Crowe and Stranks in 2018 [13]. Crowe and Stranks corroborated the findings of Barker et al. that there is an association between current [11] or previous [12] long-term BZD use and cognitive impairment. However, the findings might be difficult to generalise as most studies included in these meta-analyses were conducted on populations of problematic BZD users requiring specialised treatment, and thus the impairments measured could be related to the return of premorbid symptoms rather than reflecting the effects of chronic BZD treatment [9, 14]. In addition, these three meta-analyses address the concept of cognitive impairment indicating a cross-sectional deleterious effect of BZDs as opposed to the concept of cognitive decline which involves the longitudinal effects of BZDs on cognitive change with age [7].

Considering that the existing review [9] is somewhat out of date and no systematic review has been conducted on epidemiological studies that addressed the association between long-term BZD use and cognitive decline, the question remains open. Taken together, the potential burden of impaired cognitive functioning on society, the discrepancy of findings, and the methodological limitations of the studies, an updated review of prospective longitudinal studies addressing this issue is warranted. The aim of this review was to evaluate and integrate current data available by systematically reviewing studies that examined the association between cognitive decline and long-term exposure to BZDs in the general population.

## 2. Methods

This is a systematic literature review of prospective longitudinal studies that assessed the risk of cognitive decline in long-term BZD users compared with nonusers. It was conducted in line with the PRISMA (Preferred Reporting Items for Systematic reviews and Meta-Analyses) guidelines (Supplementary Materials [Supplementary-material supplementary-material-1]) [15].

### 2.1. Data Source

The target population was defined as the general population (adults aged 18 or older) with no restriction as to the setting. Studies involving individuals with schizophrenia or other psychotic disorder as well as those with a comorbid substance use disorder other than BZD use were excluded. These conditions have been associated with cognitive impairment and therefore might have affected the results and the generalisability of the findings [16, 17]. The exposure was long-term BZD use defined as the use of a BZD for six months or longer during a time period of one year regardless of whether the use was daily or infrequent [1]. For the purpose of this review, BZDs included the Z-drugs. The comparator (control) group consisted of individuals who were BZD nonusers. The primary outcome of interest investigated was cognitive decline assessed using cognitive function tests [11].

### 2.2. Inclusion and Exclusion Criteria

Inclusion criteria were as follows: (1) prospective longitudinal studies that investigated the effects of long-term BZD use on cognitive decline; (2) administration of cognitive tests at a minimum of two time points with no minimum period of follow-up required; (3) presence of a control group of BZD nonusers; (4) human studies with adults aged 18 years or over; (5) studies published in English in peer-reviewed journals.

The exclusion criteria were as follows: (1) retrospective or cross-sectional studies; (2) studies with children or adolescents (<18 years old); (3) studies involving participants with schizophrenia or other psychotic disorder; (4) studies involving participants with a substance use disorder other than BZD; (5) BZD use for less than six months or not specified; (6) studies that focused on the effects of withdrawing from BZD; (7) studies with dementia as the only outcome; (8) specific information about the cognitive tests utilized not included; (9) control group which included subjects with prior long-term use of BZD.

### 2.3. Data Selection

**Figure 1:**
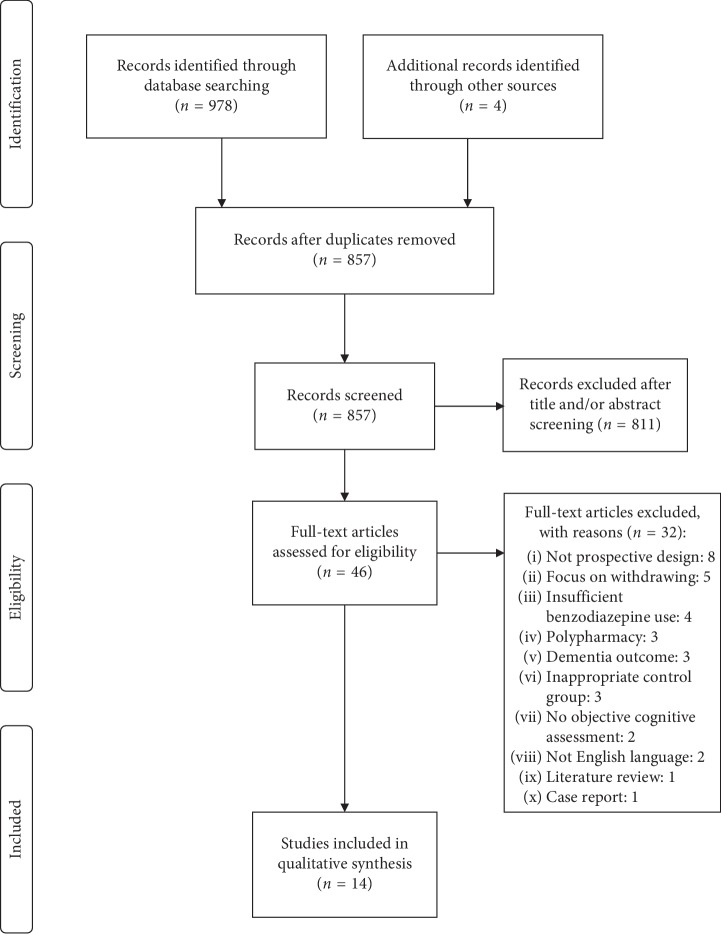
PRISMA flow diagram.

Electronic searches were conducted in the PubMed and PsycINFO databases (via APA PsycNET platform) to identify potential studies to be included in the review. The searches were carried out for scientific papers with no publication date restrictions. Searches were based on combinations of keywords related to “benzodiazepines” and “cognitive function,” with separate searches for Z-drugs (details are given in Supplementary Materials Appendices [Supplementary-material supplementary-material-1] and [Supplementary-material supplementary-material-1]). The last searches in both databases were performed on 12 February 2018. The references of the selected articles were also screened for further relevant studies. The database searches yielded a total of 978 records, of which 693 were retrieved through PubMed search (680 in Search 1; 13 in Search 2) and 285 through PsycINFO search (282 in Search 1; 3 in Search 2). One-hundred twenty-five records were identified as duplicates and removed. The remaining 853 records were initially evaluated for relevance by review of the title or abstract. Eight-hundred and eleven records met exclusion criteria and were excluded. The full text of the remaining 42 articles and of the four additional articles obtained by hand searching references were assessed against the inclusion and exclusion criteria to determine eligibility. Fourteen studies that fulfilled inclusion criteria were included in the systematic review [7, 10, 18–29].  Figure 1 shows the PRISMA flow diagram used to summarize the study selection process [15]. The search strategy was determined by both reviewers. The screening of titles, abstracts, and full texts was conducted by reviewer D.N. and approved by reviewer L.G.

### 2.4. Data Analysis

A data extraction form was developed and used to record summary information on the study design, participant characteristics, and outcomes of cognitive assessments for studies that met the criteria for inclusion. The methodological quality and risk of bias assessments of the included studies were based on the Joana Briggs Institute Critical Appraisal Checklist for Cohort Studies [30]. These assessments were conducted by reviewer D.N. and approved by reviewer L.G.

Meta-analysis was not undertaken because the combined result would not be meaningful as the outcome measures and the detail of study design of the included studies were too different. The studies differed in several key aspects such as definition of long-term BZD use, duration of BZD use, follow-up periods, cognitive domains, cognitive tests, scoring systems, and statistical analysis. Thus, a narrative synthesis [31] was conducted to provide an overview of the association between BZD use and cognitive decline.

## 3. Results

The included studies involved 2145 long-term BZD users. A description of the main features of each study can be found in [Supplementary-material supplementary-material-1] (Supplementary Materials). Also, a list of the excluded studies [14, 32–62] with reasons for exclusion is shown in [Supplementary-material supplementary-material-1] (Supplementary Materials).

### 3.1. Characteristics of Included Studies

All the studies were conducted in developed countries and had a prospective longitudinal design. They used data either from large epidemiological studies or from national databases and took place basically in outpatient setting. Except for one prospective case-control study [28], the studies were all prospective cohort studies, mostly population-based cohort studies which allow the estimation of distributions of relevant variables in a defined population [63].

Assessment of BZD use was based on self-report, pharmacist records, prescription forms, or inspection of drug containers. There was considerable variability across the studies in the described frequency of BZD from daily use to regular use or even irregular use, and there was a lack of clarity in definition of these terms. Chronic BZD use was typically defined as continued use from baseline to the follow-up endpoint and at all the measurements. It differed from previous or temporary use in not extending to the end of follow-up and from new use reported only at the follow-up endpoint. The estimated duration of use varied from 1 year to 9 years. The follow-up evaluation times ranged from 2 to 22 years and were between 3 and 10 years in 85% of the studies.

The cognitive domains were assessed by 30 cognitive tests as shown in Table 1, whereas the items that were extracted from each study such as study attributes, subject characteristics, and pattern of BZD use including frequency and estimated duration are summarised in Table 2.

### 3.2. Results of Comparisons

Only 3 of the 14 studies provided support for an association between long-term BZD use and cognitive decline [10, 20, 24]. Paterniti et al. [20] used logistic regression models to assess whether long-term BZD users were more likely to develop cognitive decline compared with nonusers. They demonstrated that long-term BZD use was significantly associated with a higher risk of decline for global cognitive functioning (OR = 1.9, 95% CI = 1.0, 3.6), psychomotor speed (OR = 2.5, 95% CI = 1.5, 4.4), and speed of processing (OR = 2.3, 95% CI = 1.3, 4.1) compared with nonusers. Further, Bierman et al. [10] performed multilevel analyses to investigate the effect of long-term BZD use on cognitive decline. They calculated the effect size (*F*^2^) and found that long-term BZD users showed decline in global cognitive functioning (*F*^2^ = 0.012, *p* < 0.001), intelligence (*F*^2^ = 0.007, *p* < 0.001), and verbal memory (delayed recall: *F*^2^ = 0.003, *p*=0.024; retention: *F*^2^ = 0.004, *p*=0.004) compared with nonusers. Finally, multivariate analyses stratified on gender by Boef-Cazou et al. [24] revealed that BZD use significantly affected verbal memory, namely, delayed free recall, but only in women. The amount of change found in women long-term BZD users compared with nonusers was indicated by the *β* value given with the standard deviation (*β* = −2.13 ± 0.67, *p* < 0.01). The results of statistical analyses from all the included studies are reported with the cognitive domains assessed, the follow-up evaluation times, and whether cognitive decline was found or not in Table 3.

On the other hand, the examination of moderators revealed that no sociodemographic variable or specific parameter of BZD use was consistently associated with an increased risk of cognitive decline. Paterniti et al. [20] found no predictors of cognitive decline. Bierman et al. [10] reported that both duration and cumulative exposure to BZD were positively correlated with cognitive decline, but duration of BZD use had a greater effect on cognitive performance than the dosage. Hanlon et al. [19] demonstrated a duration-response as well as a dose-response relationship for cognitive impairment (but not for cognitive decline) and BZD use. Conversely, other studies did not confirm this positive correlation. In the study by Gallacher et al. [26], greater duration of BZD use was not associated with greater risk of cognitive decline as participants with an estimated duration of BZD use of 4 years or less were almost twice as likely to have dementia as those with more than 4 years taking BZDs. Similarly, Gray et al. [27] reported that higher BZD use was not associated with more rapid cognitive decline. They found a small increase in the risk of dementia in people with minimal exposure to BZDs but not with the highest level of exposure.

### 3.3. Methodological Quality

The details of the appraisal tool applied to each study are shown in [Supplementary-material supplementary-material-1] (Supplementary Materials). Considering a study to be of good quality when the proportion of “Yes” answers is at least 65%, of low quality when the score is below 40%, and of moderate quality when it is between 40% and 64%, twelve studies were of moderate quality [7, 10, 18–20, 22–27, 29], one of good quality [28], and another one of low quality [21] (Table 2).

In all the studies, BZD users and nonusers were recruited from the same population and correctly assigned to their groups. Also, the confounding factors were identified as shown in Table 4. Similarly, strategies to deal with the confounders and regression analysis techniques were stated and seemed appropriate in nearly all the studies.

The most frequently identified methodological weakness was related to the BZD exposure which was not measured in a valid and reliable way in any of the studies. The measurements were determined by indirect means and typically referred to the use at interview or over short periods before ranging from 2 weeks to 2 months. In all the studies, it was assumed that the use lasted up to the following assessment and thus it provided only an estimate of the likely duration of BZD use [26]. As a result, the overall validity of the measurements was compromised. Other common methodological flaws were related to (1) baseline differences between BZD users and controls [7, 18, 20–27, 29] as shown in Table 4, (2) lack of strategies to address incomplete follow-up [7, 10, 18–27], (3) assessment of cognitive function limited to global cognitive functioning [18, 19, 23–25, 29], (4) follow-up times not considered long enough for cognitive decline to occur [19–21, 25, 28, 29], and (5) cognition not normal at baseline [7, 20–22, 26].

## 4. Discussion

### 4.1. Main Findings

This systematic review identified 14 prospective longitudinal studies addressing the association of long-term BZD use and cognitive decline. Overall, the methodological quality of the studies was similar. Twelve studies were of moderate quality, one of good quality [28], and another one of low quality [21]. The large majority of the studies reviewed failed to demonstrate a higher risk of cognitive decline in long-term BZD users. Only 3 of the 14 studies provided support for an association between long-term benzodiazepine use and cognitive decline with a small to medium effect size [10, 20, 24]. These studies were of moderate quality as well as most of the studies which failed to demonstrate a higher risk for cognitive decline. Global cognitive functioning, verbal memory, intelligence, psychomotor speed, and speed of processing were the cognitive domains affected. These three studies used different methods to assess the strength of the association between long-term BZD use and cognitive decline. The effect sizes that Bierman et al. [10] determined (*F*^2^ = 0.012, *p* < 0.001 for global cognitive functioning; *F*^2^ = 0.003, *p*=0.024, and *F*^2^ = 0.004, *p*=0.004 for verbal memory; and *F*^2^ = 0.007, *p* < 0.001 for fluid intelligence) were deemed to be small according to Cohen's convention [64] about *F*^2^ values (0.02 is small, 0.15 medium, and 0.35 large). On the other hand, Paterniti et al. [20] computed odds ratios (ORs) rather than calculating effect sizes. In order to assess the magnitude of their findings, the equivalence of OR and effect size, namely, Cohen's *d*, should be estimated. Cohen's *d*, the standardized mean difference between two group means, is generally accepted as an indication of a small (*d* = 0.2), medium (*d* = 0.5), and large (*d* = 0.8) effect size [64,65]. Based on formulas for converting between the ORs and *d*, taken from Borenstein et al. [66] and made available for use by DeCoster [67], the ORs reported by Paterniti et al. [20] were converted to *d* (global cognitive functioning: OR = 1.9, *d* = 0.35; speed of processing: OR = 2.3, *d* = 0.45; and psychomotor speed: OR = 2.5, *d* = 0.50) and corresponded to a small to medium effect size.

### 4.2. Discrepancies among the Studies

The studies in this review controlled for many, but not all, confounding factors (Table 4). Therefore, confounders might provide one possible explanation for the discrepancies among them. For instance, the three studies that found cognitive decline to be associated with long-term BZD use did not adjust for current psychiatric diagnosis or psychiatric history which could account for the cognitive decline [10, 20, 24]. Also, use of psychotropic drugs other than BZDs was either not controlled for [10, 24] or controlled for as a single variate [20] rather than considering each class of drugs separately. Therefore, the impact of a specific class of drug on cognition could have been masked [25]. Other potential confounders not adjusted for were smoking [10] and depressive and anxious symptoms [24]. Moreover, the possibility of reverse causality should be considered in these three studies as the presence of cognitive impairment due to other causes could not be ruled out and might have contributed to long-term BZD use.

### 4.3. Possible Reasons for the Negative Findings

In addition to methodological limitations which will be further addressed, the depletion of susceptible effect should be considered [68]. With regard to the BZDs, it has been postulated that physicians would stop prescribing the drug to those susceptible to, and unable to tolerate, the cognitive effects. As a result, those who kept using the BZDs would correspond to those not predisposed to develop cognitive decline nor dementia associated with long-term BZD use. The depletion of susceptible effect could not be excluded in some studies [18, 22, 25, 27].

### 4.4. Implications for Clinical Practice

Although most studies in the current review have not provided evidence of a greater risk of cognitive decline associated with long-term benzodiazepine use, there is also no strong evidence for an absence of cognitive decline either. Therefore, physicians should take a cautious approach to prescribing BZDs, especially for elderly patients, avoiding higher doses and longer duration of treatment than those generally recommended, despite the fact that a quantitative exposure-effect relationship has not yet been established.

### 4.5. Limitations

The current review was subject to several limitations. First, a selection bias might have occurred in some studies, especially with the elderly. The most debilitated ones were more prone to not be included by virtue of nonresponse or loss to follow-up (e.g., due to death), resulting in a possible underestimation of cognitive decline. Second, the definition of long-term BZD users was problematic in all the studies. It was based on measures at fixed intervals which were assumed to represent the whole period in between the follow-ups. In this way, it is possible that intermittent and chronic BZD users as well as intermittent and nonusers were mixed in the same group. Third, information on BZD use history such as dose, frequency, duration, time since last dose, and half-life was not available in most studies. Fourth, despite the relatively large cohorts, the final sample size of long-term BZD users was usually small, thus limiting the power of analysis. Moreover, the samples were nonclinical, and therefore the amount, frequency, and duration of use might not have reached the threshold for a longitudinal effect on cognitive decline. Fifth, many studies utilized cognitive tests that were more appropriate for screening gross cognitive deficits than detecting subtle neuropsychological changes over time. As a result, cognitive decline might have been underestimated. Sixth, the included studies were not able to control for all confounding factors, and the confounders that were adjusted for varied across the studies, making comparisons more difficult. Seventh, the follow-up was short (<5 years) in several studies, and thus the time frame might not have been sufficient for cognitive decline to be detected. Finally, relevant studies might have been missed due to restricting the eligibility criteria to peer-reviewed publications in English. Similarly, having no studies from developing countries, considering basic economic country conditions, might affect the generalisability of the findings.

### 4.6. Implications for Research

Future research about the long-term cognitive effects of BZDs should favor prospective studies with a case-control design (i.e., comparison of long-term users and matched nonusers). The source population should be nonclinical, given that several medical conditions may be accompanied by decrements in cognitive function. Given that the major gap in the research conducted thus far has been to find a way to better characterize degree of BZD exposure across studies, it is crucial to collect detailed information on BZD use including whether the use continued with no interruptions between the follow-up evaluations. Further, a comprehensive neuropsychological test battery should be utilized. Finally, the duration of the follow-up period should be at least 5 years which is considered a sufficient period to detect adverse outcomes associated with BZD exposure [29].

## 5. Conclusions

This systematic review found little evidence of an association between long-term BZD use and progressive cognitive decline. Only 3 of the fourteen studies demonstrated that long-term BZD use was associated with a higher risk of cognitive decline among the general adult population. However, discrepancies among the results and inconsistencies regarding the cognitive domains affected and methodological limitations involving the included studies such as selection bias, problematic definition of long-term BZD users, lack of information on BZD use history, lack of adjustment for some confounding factors, small sample size of long-term BZD users, and insufficient follow-up times in some studies prevent definite conclusions. Therefore, future research with prospective studies specially designed would be of great value.

## Figures and Tables

**Table 1:** Cognitive tests used in the studies to assess the cognitive domains.

Global cognitive functioning
Mini mental state examination [7, 10, 20, 22, 23, 25, 26]
Short portable mental status questionnaire [18, 19]
Orientation-memory-concentration test [19]
Cambridge cognitive examination [26]
Cognitive abilities screening instrument [27]
Montreal cognitive assessment [28]
Clinical dementia rating sum of boxes [29]
Intelligence
Raven's colored progressive matrices [10]
AH4 and the national adult reading test [26]
Motor control/performance
Finger tapping test [20]
Psychomotor speed
Digit symbol substitution test [20, 24]
Speed of processing
Trail making test, part B [7, 20]
Four choice reaction time task [25]
Coding task [10]
Letter digit coding test [22]
Visuospatial abilities
Matching to sample and copying tasks [21]
Verbal reasoning
Isaacs set test [7]
Visuospatial reasoning task [22]
Attention/concentration
Trail making test, part a [7]
Stroop test [22]
Selective attention test derived from the Sternberg's test [29]
Reaction time to a visual task with auditory interference [20]
Nonverbal memory
Benton visual retention test [7]
Recognition test [24]
Verbal memory
Auditory verbal learning test [10,20,28]
Immediate and delayed recall tests [21,24]
Picture learning test [22]
Object naming, verbal fluency, phoneme comprehension and syntax comprehension tasks [21]

**Table 2:** Study attributes, subject characteristics, pattern of benzodiazepine use, and risk of bias score of the studies.

Study	Country recruitment	Participants^*∗*^ (*N*)	Age (years)	Men (%)	Chronic BZD users (*n*/%)	Frequency and duration of BZD use	Total % risk of bias score
Dealberto et al. [18]	USA, random selection	1200	65–84 (97%)	34	31/2.5%	Frequency unclear 6 years	54.5
Hanlon et al. [19]	USA, random selection	2765	65–105	36	168/6.0%	Regular or as needed use 3 years	54.5
Paterniti et al. [20]	France, electoral rolls	1176	60–70	41	80/6.8%	Regular use 4 years	54.5
Allard et al. [21]	France, regional GP network	372	76 (mean)	ND	27/7.2%	Frequency unclear 3 years	36.3
Bierman et al. [10]	The Netherlands, random selection	1351	62–85	47	37/2.7%	Regular use 9 years	63.3
van Vliet et al. [22]	The Netherlands, all the town inhabitants	486	85	33	140/28.8%	Daily use 1 year	54.5
Puustinen et al. [23]	Finland, all the town inhabitants	565	64–89	40	84/14.8%	Regular or irregular use 6 years	54.5
Boeuf-Cazou et al. [24]	France, random selection	1019	32–62	52	76/7.4%	Frequency unclear 1 year	63.3
Desplenter et al. [25]	Finland, random selection	449	81 (mean)	31	139/30.9%	Regular or as needed use 1 year	54.5
Gallacher et al. [26]	Wales, random selection	1134	45–64	100	103/9.0%	Regular use 4 years	63.3
Mura et al. [7]	France, all the town inhabitants	5195	73 (mean)	40	969/18.6%	At least once a week use 2 years	63.3
Gray et al. [27]	USA, random selection	3434	70–80	40	267/7.7%	Daily use 1 year (median)	63.3
Chung et al. [28]	USA, Canada, databases	30	55–90	26	15/50.0%	Frequency unclear 6.4 years (mean)	72.7
Zhang et al. [29]	USA, databases	5423	73 (mean)	34	177/3.2%	Frequency unclear 3 years	54.5

BZD = benzodiazepine; GP = general practitioner. ^*∗*^Participants who completed the follow-up.

**Table 3:** Cognitive domains, follow-up evaluation times, and outcome measures.

Study	Cognitive domains follow-up times	Results of data analyses (95% CI)	Cognitive decline (*p* < 0.05)
Dealberto et al. [18]	GCF 6 years^*∗*^	Logistic regression: OR = 1.18 (*p*=0.848)	No
Hanlon et al. [19]	GCF 3 years	Logistic regression: *β* = 0.09 (−0.07–0.24)	No
Paterniti et al. [20]	GCF, MC, PS, SP and VM 4 years	Logistic regression: OR: GCF = 1.9 (1.0–3.6); MC = 1.2 (0.5–2.6); PS = 2.5 (1.5–4.4); SP = 2.3 (1.3–4.1); VM = 1.9 (0.8–4.5)	Yes: For GCF, PS and SP
Allard et al. [21]	AC, VM, VR and VS 3 years	ND	No
Bierman et al. [10]	GCF, I, SP and VM 9 years^*∗*^	Multilevel analyses: effect size (*F*^2^) : GCF = 0.012 (*p* < 0.001); VM (delayed recall) = 0.003 (*p*=0.024); VM (retention) = 0.004 (*p*=0.004); *I* = 0.007 (*p* < 0.001); SP = 0.005 (*p*=0.068)	Yes: For GCF, I and VM
van Vliet et al. [22]	AC, GCF, SP and VM 5 years	Linear regression analyses: difference in scores: GCF: −0.28 (−1.24–0.69) *p*=0.57; AC: 3.00 (−2.78–8.77) *p*=0.30; SP: −0.90 (−2.36–0.56) *p*=0.22; VM (immediate recall): 0.14 (−0.81–1.10) *p*=0.76; VM: (delayed recall): 0.21 (−0.22–0.64) *p*=0.34	No
Puustinen et al. [23]	GCF 7.6 years^*∗*^ (mean)	ND	No
Boeuf-Cazou et al. [24]	AC, NVM, PS and VM 10 years	Multilevel analyses: *β* scores (±SD) : men: AC: −0.30 ± 0.20; NVM: −0.57 ± 0.34 PS: −0.32 ± 1.70; VM (immediate recall): −0.40 ± 0.25 VM (delayed recall): −0.80 ± 0.42 women: AC: 0.01 ± 0.21; NVM: −0.12 ± 0.26 PS: 4.48 ± 2.65; VM (immediate recall): 0.52 ± 0.28 VM (delayed recall): −2.13 ± 0.67 (*p* < 0.01)	Yes: For VM and only for women
Desplenter et al. [25]	GCF 3 years	Linear mixed model: GCF: overall difference: 0.31 (SE = 0.16) *p*=0.051	No
Gallacher et al. [26]	GCF, I and SP 22 years	Logistic regression: OR for cognitive decline = 0.63 (0.27–1.48) *p*=0.29	No
Mura et al. [7]	AC, GCF, NVM, SP and VR 7 years	Nonlinear multivariate mixed model: interaction BZDs *x* time: AC: −0.11 (*p*=0.08); GCF: −0.06 (*p*=0.32); NVM: 0.12 (*p*=0.19); SP: 0.005 (*p*=0.97); VR: 0.05 (*p*=0.28)	No
Gray et al. [27]	GCF 7.3 years (mean)	Linear regression: Difference in the mean cognitive score: 0.002 (−0.05–0.06)	No
Chung et al. [28]	GCF, VM 2 years	Mixed-effect model: changes in the scores: GCF : F (1, 27.60) = 0.09, *p*=0.76 VM (immediate recall): *F* (1, 27.40) = 0.003, *p*=0.96; VM (% of forgetting): *F* (1, 27.63) = 0.15, *p*=0.70; VM (learning): *F* (1, 27.57) = 0.76, *p*=0.39; VM (forgetting): *F* (1, 27.63) = 0.15, *p*=0.70	No
Zhang et al. [29]	GCF 4.8 (mean)	Logistic regression: Interaction BZDs *x* time = −0.06 (*p*=0.22) for MMSE; 0.002 (*p*=0.97) for CDR-SB	No

AC = attention/concentration; BZDs = benzodiazepines; *β* = beta coefficient; CDR-SB = clinical dementia rating sum of boxes; CI = confidence interval; GCF = global cognitive functioning; I = intelligence; MC = motor control/performance; MMSE = mini mental state examination; ND = not described; NVM = nonverbal memory; OR = odds ratio; PS = psychomotor speed; SD = standard deviation; SE = standard error; SP = speed of processing; VM = verbal memory; VR = verbal reasoning; VS = visuospatial. ^*∗*^Follow-up time was not complete (less than 80% of participants were followed).

**Table 4:** Baseline differences between groups and confounding factors.

Study	Baseline differences between BZD users and nonusers	Confounding factors that were adjusted for
Dealberto et al. [18]	BZD users were more likely to have depressive symptoms	Age, gender, education, depressive symptoms, chronic diseases, psychotropic drugs, race, marital status, housing
Hanlon et al. [19]	Unclear: sample characteristics described as a whole	Age, gender, education, depressive symptoms, chronic diseases, alcohol use, smoking, race, insomnia, thyroid medication
Paterniti et al. [20]	BZD users were more likely to be older, women, use psychotropic drugs, smoke, have depressive and anxious symptoms	Age, gender, education, depressive and anxious symptoms, chronic diseases, alcohol use, smoking, psychotropic drugs
Allard et al. [21]	BZD users were older	Age, gender, education, depressive symptoms, chronic diseases, prodromal dementia
Bierman et al. [10]	Unclear: sample characteristics described as a whole	Age, gender, education, depressive and anxious symptoms, chronic diseases, alcohol use
van Vliet et al. [22]	BZD users were more likely to be women, be institutionalized, have depressive symptoms and less education	Gender, education, depressive symptoms
Puustinen et al. [23]	BZD users were more likely to be older and women	Age, gender, education, chronic diseases, smoking
Boeuf-Cazou et al. [24]	BZD users were more likely to be women	Age, education, chronic diseases, alcohol use, smoking, marital status, cognitive score at baseline, exercise, shiftwork, body mass index
Desplenter et al. [25]	BZD users were more likely to be older, women, use psychotropic drugs	Age, gender, education, depressive symptoms, use of antipsychotic drugs
Gallacher et al. [26]	BZD users were more likely to have anxious symptoms	Age, education, anxious symptoms, chronic diseases, alcohol use, smoking, social class, cognitive score at baseline, daytime sleepiness, body mass index
Mura et al. [7]	BZD users were more likely to be older, women, have depressive and cardiovascular symptoms	Gender, education, anxious, depressive and cardiovascular symptoms, chronic diseases, alcohol use, smoking, antidepressant use, insomnia, apolipoprotein E4 genotype, employment, exercise
Gray et al. [27]	Heavier BZD users were more likely to be women, have depressive and cardiovascular symptoms	Age, gender, education, depressive symptoms, chronic diseases, smoking, exercise, body mass index
Chung et al. [28]	Groups were matched on age, gender, ethnicity, education, race, apolipoprotein E4 genotype, marital status	Use of antidepressant drugs
Zhang et al. [29]	BZD users were more likely to be white, smoke, use alcohol, have cardiovascular and depressive symptoms	Age, gender, education, chronic diseases, alcohol use, smoking, race, family dementia history, brain injury

BZD = benzodiazepine.

## Supplementary Materials

Supplementary Materials Appendix S1: PRISMA checklist. Appendix S2: PubMed search strategy. Appendix S3: PsycINFO search strategy via APA PsycNET Platform. Appendix S4: characteristics of included studies. Appendix S5: characteristics of excluded studies.

## References

[1] Kurko T. A. T., Saastamoinen L. K., Tähkäpää S. (2015). Long-term use of benzodiazepines: definitions, prevalence and usage patterns—a systematic review of register-based studies. *European Psychiatry*.

[2] Lader M. (2011). Benzodiazepines revisited-will we ever learn?. *Addiction*.

[3] Dell’Osso B., Albert U., Atti A. R. (2015). Bridging the gap between education and appropriate use of benzodiazepines in psychiatric clinical practice. *Neuropsychiatric Disease and Treatment*.

[4] Verster J. C., Volkerts E. R. (2006). Clinical pharmacology, clinical efficacy, and behavioral toxicity of alprazolam: a review of the literature. *CNS Drug Reviews*.

[5] Picton J. D., Marino A. B., Nealy K. L. (2018). Benzodiazepine use and cognitive decline in the elderly. *American Journal of Health-System Pharmacy*.

[6] Stewart S. A. (2005). The effects of benzodiazepines on cognition. *The Journal of Clinical Psychiatry*.

[7] Mura T., Proust-Lima C., Akbaraly T. (2013). Chronic use of benzodiazepines and latent cognitive decline in the elderly: results from the three-city study. *European Neuropsychopharmacology*.

[8] Anstey K. J., von Sanden C., Salim A., O’Kearney R. (2007). Smoking as a risk factor for dementia and cognitive decline: a meta-analysis of prospective studies. *American Journal of Epidemiology*.

[9] Verdoux H., Lagnaoui R., Begaud B. (2005). Is benzodiazepine use a risk factor for cognitive decline and dementia? A literature review of epidemiological studies. *Psychological Medicine*.

[10] Bierman E. J. M., Comijs H. C., Gundy C. M., Sonnenberg C., Jonker C., Beekman A. T. F. (2007). The effect of chronic benzodiazepine use on cognitive functioning in older persons: good, bad or indifferent?. *International Journal of Geriatric Psychiatry*.

[11] Barker M. J., Greenwood K. M., Jackson M., Crowe S. F. (2004). Cognitive effects of long-term benzodiazepine use. *CNS Drugs*.

[12] Barker M., Greenwood K. M., Jackson M., Crowe S. F. (2004). Persistence of cognitive effects after withdrawal from long-term benzodiazepine use: a meta-analysis. *Archives of Clinical Neuropsychology*.

[13] Crowe S. F., Stranks E. K. (2018). The residual medium and long-term cognitive effects of benzodiazepine use: an updated meta-analysis. *Archives of Clinical Neuropsychology*.

[14] Deckersbach T., Moshier S. J., Tuschen-Caffier B., Otto M. W. (2011). Memory dysfunction in panic disorder: an investigation of the role of chronic benzodiazepine use. *Depression and Anxiety*.

[15] Liberati A., Altman D. G., Tetzlaff J. (2009). The PRISMA statement for reporting systematic reviews and meta-analyses of studies that evaluate health care interventions: explanation and elaboration. *PLoS Med*.

[16] Sheffield J. M., Barch D. M. (2016). Cognition and resting-state functional connectivity in schizophrenia. *Neuroscience & Biobehavioral Reviews*.

[17] Bruijnen C. J. W. H., Dijkstra B. A. G., Walvoort S. J. W. (2019). Prevalence of cognitive impairment in patients with substance use disorder. *Drug and Alcohol Review*.

[18] Dealberto M.-J., Mcavay G. J., Seeman T., Berkman L. (1997). Psychotropic drug use and cognitive decline among older men and women. *International Journal of Geriatric Psychiatry*.

[19] Hanlon J. T., Horner R. D., Schmader K. E. (1998). Benzodiazepine use and cognitive function among community-dwelling elderly. *Clinical Pharmacology & Therapeutics*.

[20] Paterniti S., Dufouil C., Alpérovitch A. (2002). Long-term benzodiazepine use and cognitive decline in the elderly: the epidemiology of vascular aging study. *Journal of Clinical Psychopharmacology*.

[21] Allard J., Artero S., Ritchie K. (2003). Consumption of psychotropic medication in the elderly: a re-evaluation of its effect on cognitive performance. *International Journal of Geriatric Psychiatry*.

[22] van Vliet P., van der Mast R. C., van den Broek M., Westendorp R. G. J., de Craen A. J. M. (2009). Use of benzodiazepines, depressive symptoms and cognitive function in old age. *International Journal of Geriatric Psychiatry*.

[23] Puustinen J., Nurminen J., Löppönen M. (2011). Use of CNS medications and cognitive decline in the aged: a longitudinal population-based study. *BMC Geriatrics*.

[24] Boeuf-Cazou O., Bongue B., Ansiau D., Marquié J.-C., Lapeyre-Mestre M. (2011). Impact of long-term benzodiazepine use on cognitive functioning in young adults: the VISAT cohort. *European Journal of Clinical Pharmacology*.

[25] Desplenter F., Lavikainen P., Hartikainen S., Sulkava R., Bell J. S. (2012). Sedative use and incident cognitive decline among persons aged 75 years and older: a population-based longitudinal study. *International Psychogeriatrics*.

[26] Gallacher J., Elwood P., Pickering J., Bayer A., Fish M., Ben-Shlomo Y. (2012). Benzodiazepine use and risk of dementia: evidence from the Caerphilly Prospective Study (CaPS). *Journal of Epidemiology and Community Health*.

[27] Gray S. L., Dublin S., Yu O. (2016). Benzodiazepine use and risk of incident dementia or cognitive decline: prospective population based study. *BMJ*.

[28] Chung J. K., Nakajima S., Shinagawa S. (2016). Benzodiazepine use attenuates cortical *β*-amyloid and is not associated with progressive cognitive decline in nondemented elderly adults: a pilot study using F18-florbetapir positron emission tomography. *The American Journal of Geriatric Psychiatry*.

[29] Zhang Y., Zhou X.-H., Meranus D. H., Wang L., Kukull W. A. (2016). Benzodiazepine use and cognitive decline in elderly with normal cognition. *Alzheimer Disease & Associated Disorders*.

[30] The Joana Briggs Institute (2019). Joana Briggs Institute 2017 critical appraisal checklist for cohort studies. http://joannabriggs.org/research/critical-appraisal-tools.html.

[31] Ryan R. (2016). Cochrane consumers and communication review group. Cochrane consumers and communication review group: data synthesis and analysis. http://cccrg.cochrane.org.

[32] Hendler N., Cimini C., Ma T., Long D. (1980). A comparison of cognitive impairment due to benzodiazepines and to narcotics. *The American Journal of Psychiatry*.

[33] Lucki I., Rickels K. (1986). The behavioral effects of benzodiazepines following long-term use. *Psychopharmacology Bulletin*.

[34] Golombok S., Moodley P., Lader M. (1988). Cognitive impairment in long-term benzodiazepine users. *Psychological Medicine*.

[35] Salzman C., Fisher J., Nobel K., Glassman R., Wolfson A., Kelley M. (1992). Cognitive improvement following benzodiazepine discontinuation in elderly nursing home residents. *International Journal of Geriatric Psychiatry*.

[36] Tata P. R., Rollings J., Collins M., Pickering A., Jacobson R. R. (1994). Lack of cognitive recovery following withdrawal from long-term benzodiazepine use. *Psychological Medicine*.

[37] Gorenstein C., Bernik M. A., Pompéia S., Marcourakis T. (1995). Impairment of performance associated with long-term use of benzodiazepines. *Journal of Psychopharmacology*.

[38] Foy A., O’Connell D., Henry D., Kelly J., Cocking S., Halliday J. (1995). Benzodiazepine use as a cause of cognitive impairment in elderly hospital inpatients. *The Journals of Gerontology Series A: Biological Sciences and Medical Sciences*.

[39] Berg S., Dellasega C. (1996). The use of psychoactive medications and cognitive function in older adults. *Journal of Aging and Health*.

[40] Fastbom J., Forsell Y., Winblad B. (1998). Benzodiazepines may have protective effects against Alzheimer disease. *Alzheimer Disease & Associated Disorders*.

[41] Sumner D. D. (1998). Benzodiazepine-induced persisting amnestic disorder: are older aduls at risk?. *Archives of Psychiatric Nursing*.

[42] Rickels K., Lucki I., Schweizer E., Garcia-Espana F., Case W. G. (1999). Psychomotor performance of long-term benzodiazepine users before, during, and after benzodiazepine discontinuation. *Journal of Clinical Psychopharmacology*.

[43] Vignola A., Lamoureux C., Bastien C. H., Morin C. M. (2000). Effects of chronic insomnia and use of benzodiazepines on daytime performance in older adults. *The Journals of Gerontology Series B: Psychological Sciences and Social Sciences*.

[44] Gagné A., Morin C. M. (2001). Predicting treatment response in older adults with insomnia. *Journal of Clinical Geropsychology*.

[45] Lagnaoui R., Begaud B., Moore N. (2002). Benzodiazepine use and risk of dementia: a nested case-control study. *Journal of Clinical Epidemiology*.

[46] Wadsworth E. J. K., Moss S. C., Simpson S. A., Smith A. P. (2003). Preliminary investigation of the association between psychotropic medication use and accidents, minor injuries and cognitive failures. *Human Psychopharmacology: Clinical and Experimental*.

[47] Curran H. V., Collins R., Fletcher S., Kee S. C. Y., Woods B., Iliffe S. (2003). Older adults and withdrawal from benzodiazepine hypnotics in general practice: effects on cognitive function, sleep, mood and quality of life. *Psychological Medicine*.

[48] McAndrews M. P., Weiss R. T., Sandor P., Taylor A., Carlen P. L., Shapiro C. M. (2003). Cognitive effects of long‐term benzodiazepine use in older adults. *Human Psychopharmacology*.

[49] Nyström C. (2005). Effects of long-term benzodiazepine medication. A prospective cohort study: methodological and clinical aspects. *Nordic Journal of Psychiatry*.

[50] Barker M. J., Greenwood K. M., Jackson M., Crowe S. F. (2005). An evaluation of persisting cognitive effects after withdrawal from long-term benzodiazepine use. *Journal of the International Neuropsychological Society*.

[51] Puustinen J., Nurminen J., Kukola M., Vahlberg T., Laine K., Kivelä S.-L. (2007). Associations between use of benzodiazepines or related drugs and health, physical abilities and cognitive function. *Drugs & Aging*.

[52] Bicca M. G., de Lima Argimon I. I. (2008). Habilidades cognitivas e uso de benzodiazepínicos em idosas institucionalizadas. *Jornal Brasileiro de Psiquiatria*.

[53] Wright R. M., Roumani Y. F., Boudreau R. (2009). Effect of central nervous system medication use on decline in cognition in community-dwelling older adults: findings from the health, aging and body composition study. *Journal of the American Geriatrics Society*.

[54] Lagnaoui R., Tournier M., Moride Y. (2008). The risk of cognitive impairment in older community-dwelling women after benzodiazepine use. *Age and Ageing*.

[55] Tsunoda K., Uchida H., Suzuki T., Watanabe K., Yamashima T., Kashima H. (2010). Effects of discontinuing benzodiazepine-derivative hypnotics on postural sway and cognitive functions in the elderly. *International Journal of Geriatric Psychiatry*.

[56] Gnjidic D., Le Couteur D. G., Naganathan V. (2012). Effects of drug burden index on cognitive function in older men. *Journal of Clinical Psychopharmacology*.

[57] Høiseth G., Tanum L., Tveito M. (2013). A clinical study of the cognitive effects of benzodiazepines in psychogeriatric patients. *Pharmacopsychiatry*.

[58] Farrell B., Eisener-Parsche P., Dalton D. (2014). Turning over the rocks: role of anticholinergics and benzodiazepines in cognitive decline and falls. *Canadian Family Physician Medecin de Famille Canadien*.

[59] Tveito M., Lorentzen B., Engedal K. (2014). Changes in cognitive function during psychogeriatric treatment in relation to benzodiazepine cessation. *Pharmacopsychiatry*.

[60] Bourgeois J., Elseviers M. M., Van Bortel L., Petrovic M., Vander Stichele R. H. (2015). The impact of chronic benzodiazepine use on cognitive evolution in nursing home residents. *Human Psychopharmacology: Clinical and Experimental*.

[61] Helmes E., Ø lmes T. (2015). Associations between benzodiazepine use and neuropsychological test scores in older adults. *Canadian Journal on Aging*.

[62] Federico A., Tamburin S., Maier A. (2017). Multifocal cognitive dysfunction in high-dose benzodiazepine users: a cross-sectional study. *Neurological Sciences*.

[63] Szklo M. (1998). Population-based cohort studies. *Epidemiologic Reviews*.

[64] Cohen J. (1988). *Statistical Power Analysis for the Behavioral Sciences*.

[65] Chen H., Cohen P., Chen S. (2010). How big is a big odds ratio? Interpreting the magnitudes of odds ratios in epidemiological studies. *Communications in Statistics—Simulation and Computation*.

[66] Borenstein M., Hedges L. V., Higgins J. P., Rothstein H. R. (2009). *Introduction to Meta-Analysis*.

[67] DeCoster J. (2019). Converting effect sizes. http://stat-help.com/.

[68] Yola M., Lucien A. (1994). Evidence of the depletion of susceptibles effect in non-experimental pharmacoepidemiologic research. *Journal of Clinical Epidemiology*.

