# Supplementary material for: Is Long-Term Benzodiazepine Use a Risk Factor for Cognitive Decline? Results of a Systematic Review
Source: J Addict. 2020 Jan 23;2020:1569456. doi: 10.1155/2020/1569456 (PMC7001667; doi:10.1155/2020/1569456)
Supplement: Supplementary Materials — Appendix S1: PRISMA checklist. Appendix S2: PubMed search strategy. Appendix S3: PsycINFO search strategy via APA PsycNET Platform. Appendix S4: characteristics of included studies. Appendix S5: characteristics of excluded studies. [file 1569456.f1.docx]

**Supplementary Materials**

**Appendix S1. PRISMA Checklist**

| **Section/topic** | **#** | **Checklist item** | **Reported on page #** |
| --- | --- | --- | --- |
| **TITLE** | | |  |
| Title | 1 | Identify the report as a systematic review, meta-analysis, or both. | 1 |
| **ABSTRACT** | | |  |
| Structured summary | 2 | Provide a structured summary including, as applicable: background; objectives; data sources; study eligibility criteria, participants, and interventions; study appraisal and synthesis methods; results; limitations; conclusions and implications of key findings; systematic review registration number. | 2 |
| **INTRODUCTION** | | |  |
| Rationale | 3 | Describe the rationale for the review in the context of what is already known. | 3-4 |
| Objectives | 4 | Provide an explicit statement of questions being addressed with reference to participants, interventions, comparisons, outcomes, and study design (PICOS). | 4 |
| **METHODS** | | |  |
| Protocol and registration | 5 | Indicate if a review protocol exists, if and where it can be accessed (e.g., Web address), and, if available, provide registration information including registration number. | N/A |
| Eligibility criteria | 6 | Specify study characteristics (e.g., PICOS, length of follow-up) and report characteristics (e.g., years considered, language, publication status) used as criteria for eligibility, giving rationale. | 4-5 |
| Information sources | 7 | Describe all information sources (e.g., databases with dates of coverage, contact with study authors to identify additional studies) in the search and date last searched. | 5 |
| Search | 8 | Present full electronic search strategy for at least one database, including any limits used, such that it could be repeated. | Appendices  S2 & S3 |
| Study selection | 9 | State the process for selecting studies (i.e., screening, eligibility, included in systematic review, and, if applicable, included in the meta-analysis). | 5 |
| Data collection process | 10 | Describe method of data extraction from reports (e.g., piloted forms, independently, in duplicate) and any processes for obtaining and confirming data from investigators. | 5-6 |
| Data items | 11 | List and define all variables for which data were sought (e.g., PICOS, funding sources) and any assumptions and simplifications made. | 4 |
| Risk of bias in individual studies | 12 | Describe methods used for assessing risk of bias of individual studies (including specification of whether this was done at the study or outcome level), and how this information is to be used in any data synthesis. | 6 |
| Summary measures | 13 | State the principal summary measures (e.g., risk ratio, difference in means). | N/A |
| Synthesis of results | 14 | Describe the methods of handling data and combining results of studies, if done, including measures of consistency (e.g., I^2^) for each meta-analysis. | 6 |

| **Section/topic** | **#** | **Checklist item** | **Reported on page #** |
| --- | --- | --- | --- |
| Risk of bias across studies | 15 | Specify any assessment of risk of bias that may affect the cumulative evidence (e.g., publication bias, selective reporting within studies). | N/A |
| Additional analyses | 16 | Describe methods of additional analyses (e.g., sensitivity or subgroup analyses, meta-regression), if done, indicating which were pre-specified. | N/A |
| **RESULTS** | | |  |
| Study selection | 17 | Give numbers of studies screened, assessed for eligibility, and included in the review, with reasons for exclusions at each stage, ideally with a flow diagram. | 5 |
| Study characteristics | 18 | For each study, present characteristics for which data were extracted (e.g., study size, PICOS, follow-up period) and provide the citations. | 6-7 & Tables 1 & 2 |
| Risk of bias within studies | 19 | Present data on risk of bias of each study and, if available, any outcome level assessment (see item 12). | 8 & Table 2 & Appendix S4 |
| Results of individual studies | 20 | For all outcomes considered (benefits or harms), present, for each study: (a) simple summary data for each intervention group (b) effect estimates and confidence intervals, ideally with a forest plot. | 7-8 & Table 3 & Appendix S4 |
| Synthesis of results | 21 | Present results of each meta-analysis done, including confidence intervals and measures of consistency. | 7-8 & Table 3 |
| Risk of bias across studies | 22 | Present results of any assessment of risk of bias across studies (see Item 15). | N/A |
| Additional analysis | 23 | Give results of additional analyses, if done (e.g., sensitivity or subgroup analyses, meta-regression [see Item 16]). | N/A |
| **DISCUSSION** | | |  |
| Summary of evidence | 24 | Summarize the main findings including the strength of evidence for each main outcome; consider their relevance to key groups (e.g., healthcare providers, users, and policy makers). | 9-10 |
| Limitations | 25 | Discuss limitations at study and outcome level (e.g., risk of bias), and at review-level (e.g., incomplete retrieval of identified research, reporting bias). | 10-11 |
| Conclusions | 26 | Provide a general interpretation of the results in the context of other evidence, and implications for future research. | 11-12 |
| **FUNDING** | | |  |
| Funding | 27 | Describe sources of funding for the systematic review and other support (e.g., supply of data); role of funders for the systematic review. | N/A |

**Appendix S2. PubMed search strategy**

**Search 1**

1.“Benzodiazepines” [MeSH Terms]

2. Benzodiazepine misuse [Title/abstract]

3. Benzodiazepine abuse [Title/abstract]

4. Benzodiazepine dependence [Title/abstract]

5. “Anti-anxiety agents” [MeSH Terms]

6. “Hypnotics and sedatives” [MeSH Terms]

7. “Tranquilizing agents” [MeSH Terms]

8. #1 OR #2 OR #3 OR #4 OR #5 OR #6 OR #7

9. Cognitive decline [Title/abstract]

10. Cognitive disorder [Title/abstract]

11. Cognitive function [Title/abstract]

12. #9 OR #10 OR #11

13. #8 AND #12

14. "Humans" [MeSH Terms]

15. English [Language]

16. #13 AND #14 AND #15

**Search 2**

1. “Z-drugs” [All Fields]

2. "Zolpidem"[Supplementary Concept] OR "zolpidem"[All Fields]

3. "Zaleplon"[Supplementary Concept] OR "zaleplon"[All Fields]

4. “Zopiclone"[Supplementary Concept] OR "zopiclone"[All Fields]

5. "Eszopiclone"[MeSH Terms] OR "eszopiclone"[All Fields]

6. #1 OR #2 OR #3 OR #4 OR #5

7. Cognitive decline [Title/abstract]

8. Cognitive disorder [Title/abstract]

9. Cognitive function [Title/abstract]

10. #7 OR #8 OR #9

11. #6 AND #10

12. "Humans" [MeSH Terms]

13. English [Language]

14. #11 AND #12 AND #13

**Appendix S3. PsycINFO search strategy via APA PsycNET Platform**

**Search 1**

Index Terms: {Benzodiazepines} OR {Hypnotic Drugs} OR {Sedatives} OR {Minor Tranquilizers} OR {Tranquilizing Drugs}

AND Index Terms: {Cognition} OR {Cognitive Impairment} OR {Cognitive Ability} OR {Cognitive Control} OR {Cognitive Processes} OR {Executive Function} OR {Memory Disorders}

AND no filters were applied

**Search 2**

Any Field: z-drugs OR Any Field: zolpidem OR Any Field: zaleplon OR Any Field: zopiclone OR Any Field: eszopiclone

AND Index Terms: {Cognition} OR {Cognitive Impairment} OR {Cognitive Ability} OR {Cognitive Control} OR {Cognitive Processes} OR {Executive Function} OR {Memory Disorders}

AND no filters were applied

**Appendix S4. Characteristics of included studies**

**Dealberto 1997 [18]**

| Methods | Prospective cohort study, population-based |
| --- | --- |
| Participants | Setting: outpatient, participants aged 65 and over interviewed at home  Source: EPESE Study, New Haven site, USA  N = 1200 subjects with normal cognition at baseline who completed the 6-year follow-up  71.5% under 75 years old, 34.6% men, 37.6% married, 40% less than 8 years of schooling, and 12.4% had depressive symptomatology  6.1% used benzodiazepines (BZDs) at baseline and 2.2% at follow-up |
| Assessments | Exposure to BZDs defined as use in the past 2 weeks: (1) no use; (2) continuous use; (3) temporary use, intake reported only at baseline; and (4) new use, intake reported only in follow-up  Cognitive test at baseline and at follow-up: Short Portable Mental Status Questionnaire (SPMSQ) |
| Outcomes | Cognitive decline assessed by change in SPMSQ scores |
| Notes | EPESE: Established Populations for Epidemiologic Studies in the Elderly |

| ***Critical Appraisal*** | **Yes** | **No** | **Unclear** | **Not applicable** |
| --- | --- | --- | --- | --- |
| 1. Were the two groups similar and recruited from the same population? |  | x |  |  |
| 2. Were the exposures measured similarly to assign people to both exposed and unexposed groups? | x |  |  |  |
| 3. Was the exposure measured in a valid and reliable way? |  | x |  |  |
| 4. Were confounding factors identified? | x |  |  |  |
| 5. Were strategies to deal with confounding factors stated? | x |  |  |  |
| 6. Were the groups/participants free of the outcome at the start of the study (or at the moment of exposure)? | x |  |  |  |
| 7. Were the outcomes measured in a valid and reliable way? |  | x |  |  |
| 8. Was the follow up time reported and sufficient to be long enough for outcomes to occur? | x |  |  |  |
| 9. Was follow up complete, and if not, were the reasons to loss to follow up described and explored? |  | x |  |  |
| 10. Were strategies to address incomplete follow up utilized? |  | x |  |  |
| 11. Was appropriate statistical analysis used? | x |  |  |  |

**Hanlon 1998 [19]**

| Methods | Prospective cohort study, population-based |
| --- | --- |
| Participants | Setting: outpatient, participants aged 65 and over interviewed at home  Source: EPESE Study, Duke site, USA  N = 2765 subjects cognitively intact at baseline who completed the 3-year follow-up  69.7% under 75 years old, 36.1% men, 35.3% were Black, 33.1% less than 8 years of schooling, and 8.7% had severe depressive symptoms  4.7% used benzodiazepines (BZDs) at baseline and 9.5% at follow-up |
| Assessments | Exposure to BZDs defined as use in the past 2 weeks: (1) current users (use at second in-person interview); (2) previous users (use at first in- person interview only); and (3) non-users (no BZD use initially or 3 years later). Current users were further categorized as long-term users (use at both first and second interviews) or short-term users (use at second interview only)  Cognitive test at baseline and at follow-up: Short Portable Mental Status Questionnaire (SPMSQ) |
| Outcomes | Cognitive decline assessed by change in SPMSQ scores |
| Notes | EPESE: Established Populations for Epidemiologic Studies in the Elderly  Orientation-Memory-Concentration Test was also administered at follow-up to evaluate cognitive impairment |

| ***Critical Appraisal*** | **Yes** | **No** | **Unclear** | **Not applicable** |
| --- | --- | --- | --- | --- |
| 1. Were the two groups similar and recruited from the same population? |  |  | x |  |
| 2. Were the exposures measured similarly to assign people to both exposed and unexposed groups? | x |  |  |  |
| 3. Was the exposure measured in a valid and reliable way? |  | x |  |  |
| 4. Were confounding factors identified? | x |  |  |  |
| 5. Were strategies to deal with confounding factors stated? | x |  |  |  |
| 6. Were the groups/participants free of the outcome at the start of the study (or at the moment of exposure)? | x |  |  |  |
| 7. Were the outcomes measured in a valid and reliable way? |  | x |  |  |
| 8. Was the follow up time reported and sufficient to be long enough for outcomes to occur? |  | x |  |  |
| 9. Was follow up complete, and if not, were the reasons to loss to follow up described and explored? | x |  |  |  |
| 10. Were strategies to address incomplete follow up utilized? |  | x |  |  |
| 11. Was appropriate statistical analysis used? | x |  |  |  |

**Paterniti 2002 [20]**

| Methods | Prospective cohort study, population-based |
| --- | --- |
| Participants | Setting: outpatient, participants aged 60 to 70 years interviewed at the study center  Source: EVA Study, France  N = 1176 subjects participated in the 2-year and 4-year follow-up examinations  Mean age of the benzodiazepine (BZD) users (13.5%): 65.3 years; non- users (86.5%): 65 years; male gender: 23.7% of users and 44.4% of the non-users.  Years of schooling (mean): users 10.3; non-users 10.9.  BZD users had higher depressive scores than non-users (p = 0.0001) |
| Assessments | Exposure to BZDs defined as use in the last month defined: (1) non-users; (2) episodic users; (3) recurrent users; and (4) chronic users  Cognitive tests at baseline and at follow-ups: Mini Mental State Examination, Trail Making Test, part B, Digit Symbol Substitution test, Auditory Verbal Learning Test and Finger Tapping Test |
| Outcomes | Cognitive decline assessed by change in cognitive scores |
| Notes | EVA: Epidemiology of Vascular Aging study |

| ***Critical Appraisal*** | **Yes** | **No** | **Unclear** | **Not applicable** |
| --- | --- | --- | --- | --- |
| 1. Were the two groups similar and recruited from the same population? |  | x |  |  |
| 2. Were the exposures measured similarly to assign people to both exposed and unexposed groups? | x |  |  |  |
| 3. Was the exposure measured in a valid and reliable way? |  | x |  |  |
| 4. Were confounding factors identified? | x |  |  |  |
| 5. Were strategies to deal with confounding factors stated? | x |  |  |  |
| 6. Were the groups/participants free of the outcome at the start of the study (or at the moment of exposure)? |  | x |  |  |
| 7. Were the outcomes measured in a valid and reliable way? | x |  |  |  |
| 8. Was the follow up time reported and sufficient to be long enough for outcomes to occur? |  | x |  |  |
| 9. Was follow up complete, and if not, were the reasons to loss to follow up described and explored? | x |  |  |  |
| 10. Were strategies to address incomplete follow up utilized? |  | x |  |  |
| 11. Was appropriate statistical analysis used? | x |  |  |  |

**Allard 2003 [21]**

| Methods | Prospective cohort study, population-based |
| --- | --- |
| Participants | Setting: mostly outpatient (but a representative sample of institutionalized elderly was included), participants were given a test battery at home  Source: Eugeria Study, Canada  N = 372 subjects followed through three waves (T1 to T3) at yearly intervals  13.2% used a psychotropic medication across the three waves, mean age of the users (77.1 y) was significantly higher than non-users (74.2 y), no signiﬁcant difference was found between users and non-users regarding gender or education level, 7.5% reported chronic benzodiazepine (BZD) use |
| Assessments | Exposure to BZDs defined as: (1) non-consumers; (2) chronic consumers (those taking medication at T1 and T3); (3) transient consumers (those using medication at T1 but not at T3); (4) new consumers (those who did not take medication at T1 but did so at T3)  Cognitive tests at baseline and at follow-ups: reaction time to a visual task with auditory interference, immediate and delayed recall, matching to sample and copying tasks, object naming, verbal ﬂuency, phoneme comprehension and syntax comprehension tasks, and a visuospatial reasoning task |
| Outcomes | Cognitive decline assessed by change in cognitive scores, and to what extent such cognitive changes might be attributable to psychotropic use or other factors, notably age and co-morbidity |
| Notes | The aim of the study was to examine the relationship between the use of psychotropic drugs in general and cognitive performance.  BZDs were included along with other drugs. |

| ***Critical Appraisal*** | **Yes** | **No** | **Unclear** | **Not applicable** |
| --- | --- | --- | --- | --- |
| 1. Were the two groups similar and recruited from the same population? |  | x |  |  |
| 2. Were the exposures measured similarly to assign people to both exposed and unexposed groups? | x |  |  |  |
| 3. Was the exposure measured in a valid and reliable way? |  | x |  |  |
| 4. Were confounding factors identified? | x |  |  |  |
| 5. Were strategies to deal with confounding factors stated? | x |  |  |  |
| 6. Were the groups/participants free of the outcome at the start of the study (or at the moment of exposure)? |  | x |  |  |
| 7. Were the outcomes measured in a valid and reliable way? |  |  | x |  |
| 8. Was the follow up time reported and sufficient to be long enough for outcomes to occur? |  | x |  |  |
| 9. Was follow up complete, and if not, were the reasons to loss to follow up described and explored? | x |  |  |  |
| 10. Were strategies to address incomplete follow up utilized? |  | x |  |  |
| 11. Was appropriate statistical analysis used? |  |  | x |  |

**Bierman 2007 [10]**

| Methods | Prospective cohort study, population-based |
| --- | --- |
| Participants | Setting: outpatient, participants aged 62 and over interviewed at home  Source: LASA Study, The Netherlands  N = 1351 subjects in the 4^th^ measurement (T3)  Mean age 69.1 years, and 47.5% men at baseline; 32% reported benzodiazepine (BZD) use at least in one of the four assessments over the 9-year follow-up. 7.5% of the users were chronic users. |
| Assessments | Chronic BZD use was defined as BZD use at T0, T1, T2, and T3; intermittent users were excluded from analyses  Cognitive tests at baseline and at follow-ups: Mini Mental State Examination, Raven’s Colored Progressive Matrices, Coding Task, and Auditory Verbal Learning Test |
| Outcomes | Cognitive decline assessed by change in cognitive scores |
| Notes | LASA: Longitudinal Aging Study Amsterdam  The use of BZDs, type, dosage and frequency were assessed at all 4 measurements resulting in a cumulative exposure for each respondent |

| ***Critical Appraisal*** | **Yes** | **No** | **Unclear** | **Not applicable** |
| --- | --- | --- | --- | --- |
| 1. Were the two groups similar and recruited from the same population? |  |  | x |  |
| 2. Were the exposures measured similarly to assign people to both exposed and unexposed groups? | x |  |  |  |
| 3. Was the exposure measured in a valid and reliable way? |  | x |  |  |
| 4. Were confounding factors identified? | x |  |  |  |
| 5. Were strategies to deal with confounding factors stated? | x |  |  |  |
| 6. Were the groups/participants free of the outcome at the start of the study (or at the moment of exposure)? |  |  | x |  |
| 7. Were the outcomes measured in a valid and reliable way? | x |  |  |  |
| 8. Was the follow up time reported and sufficient to be long enough for outcomes to occur? | x |  |  |  |
| 9. Was follow up complete, and if not, were the reasons to loss to follow up described and explored? | x |  |  |  |
| 10. Were strategies to address incomplete follow up utilized? |  | x |  |  |
| 11. Was appropriate statistical analysis used? | x |  |  |  |

**van Vliet 2009 [22]**

| Methods | Prospective cohort study, population-based |
| --- | --- |
| Participants | Setting: outpatient, participants aged 85 interviewed at home  Source: Leiden 85-plus Study, The Netherlands  N = 599 but pharmacist data at baseline were not available for 77 subjects who were more likely to live institutionalized  Benzodiazepine (BZD) use varied between 27% and 33% during the 5-year follow-up  BZD users were more likely to be female, be institutionalized, have a low level of education, and have depressive symptoms. |
| Assessments | Exposure to BZD defined as users versus non-users. BZD users: use at least on one birthday on a daily basis in the 2 months before and 2 months after; infrequent BZD users (those who used less than once a day) were not included in the analysis  Cognitive tests at baseline and at follow-ups: Mini Mental State Examination, Stroop Test, Letter Digit Coding Test, and Picture Learning Test |
| Outcomes | Cognitive decline assessed by change in cognitive scores (cognitive function over time assessed by repeated measurements of various domains) |
| Notes |  |

| ***Critical Appraisal*** | **Yes** | **No** | **Unclear** | **Not applicable** |
| --- | --- | --- | --- | --- |
| 1. Were the two groups similar and recruited from the same population? |  | x |  |  |
| 2. Were the exposures measured similarly to assign people to both exposed and unexposed groups? | x |  |  |  |
| 3. Was the exposure measured in a valid and reliable way? |  | x |  |  |
| 4. Were confounding factors identified? | x |  |  |  |
| 5. Were strategies to deal with confounding factors stated? |  | x |  |  |
| 6. Were the groups/participants free of the outcome at the start of the study (or at the moment of exposure)? |  | x |  |  |
| 7. Were the outcomes measured in a valid and reliable way? | x |  |  |  |
| 8. Was the follow up time reported and sufficient to be long enough for outcomes to occur? | x |  |  |  |
| 9. Was follow up complete, and if not, were the reasons to loss to follow up described and explored? | x |  |  |  |
| 10. Were strategies to address incomplete follow up utilized? |  | x |  |  |
| 11. Was appropriate statistical analysis used? | x |  |  |  |

**Puustinen 2011 [23]**

| Methods | Prospective cohort study, population-based |
| --- | --- |
| Participants | Setting: outpatient, participants aged 64 and over interviewed at a health center  Source: Lieto Study, Finland  N = 565 cognitively intact at baseline completed the follow-up (mean 7.6 years; range 6.4 - 9.1 years)  77.7% aged 64 to74 years, 22.3% 75 years or older, 40% men, 63.2% married, 85.7% had basic education  Use of benzodiazepines (BZDs): 20% at baseline, 32% at follow-up, more common in women and among the older age group  73% of BZD users at baseline continued to use at follow-up |
| Assessments | Exposure to BZD was dichotomized (regular or irregular use vs. no use) Cognitive test at baseline and at follow-up: Mini Mental State Examination (MMSE) |
| Outcomes | Cognitive decline assessed by change in MMSE scores |
| Notes |  |

| ***Critical Appraisal*** | **Yes** | **No** | **Unclear** | **Not applicable** |
| --- | --- | --- | --- | --- |
| 1. Were the two groups similar and recruited from the same population? |  | x |  |  |
| 2. Were the exposures measured similarly to assign people to both exposed and unexposed groups? | x |  |  |  |
| 3. Was the exposure measured in a valid and reliable way? |  | x |  |  |
| 4. Were confounding factors identified? | x |  |  |  |
| 5. Were strategies to deal with confounding factors stated? | x |  |  |  |
| 6. Were the groups/participants free of the outcome at the start of the study (or at the moment of exposure)? | x |  |  |  |
| 7. Were the outcomes measured in a valid and reliable way? |  | x |  |  |
| 8. Was the follow up time reported and sufficient to be long enough for outcomes to occur? | x |  |  |  |
| 9. Was follow up complete, and if not, were the reasons to loss to follow up described and explored? | x |  |  |  |
| 10. Were strategies to address incomplete follow up utilized? |  | x |  |  |
| 11. Was appropriate statistical analysis used? |  |  | x |  |

**Boeuf-Cazou 2011 [24]**

| Methods | Prospective cohort study |
| --- | --- |
| Participants | Setting: outpatient, participants were current and former salaried workers, interviewed at 3-time points during the compulsory medical examination by the occupational physician,  Source: VISAT Study, France  N = 1019 subjects who completed the 10-year follow-up  Aged 32, 42, 52 and 62 years, 52.6% men  3.9% of subjects were defined as occasional users of benzodiazepine (BZD) and 7.5% as long-term users |
| Assessments | Exposure to BZDs was defined: (1) non-users; (2) occasional users, reported use in one survey or for less than 1 year; (3) long-term users, reported use in two or three successive surveys or for at least 1 year.  Cognitive tests at baseline and at follow-ups: Auditory Verbal Learning Test, the Digit Symbol Substitution Test, a selective attention test derived from the Sternberg’s test, a delayed recall test, and a recognition test |
| Outcomes | Cognitive decline according to gender and assessed by change in cognitive scores |
| Notes | VISAT: Aging, Health and Work study  Not strictly population-based as the study population was limited to salaried workers still working or just retired |

| ***Critical Appraisal*** | **Yes** | **No** | **Unclear** | **Not applicable** |
| --- | --- | --- | --- | --- |
| 1. Were the two groups similar and recruited from the same population? |  | x |  |  |
| 2. Were the exposures measured similarly to assign people to both exposed and unexposed groups? | x |  |  |  |
| 3. Was the exposure measured in a valid and reliable way? |  | x |  |  |
| 4. Were confounding factors identified? | x |  |  |  |
| 5. Were strategies to deal with confounding factors stated? | x |  |  |  |
| 6. Were the groups/participants free of the outcome at the start of the study (or at the moment of exposure)? |  |  | x |  |
| 7. Were the outcomes measured in a valid and reliable way? | x |  |  |  |
| 8. Was the follow up time reported and sufficient to be long enough for outcomes to occur? | x |  |  |  |
| 9. Was follow up complete, and if not, were the reasons to loss to follow up described and explored? | x |  |  |  |
| 10. Were strategies to address incomplete follow up utilized? |  | x |  |  |
| 11. Was appropriate statistical analysis used? | x |  |  |  |

**Desplenter 2012 [25]**

| Methods | Prospective cohort study, population-based |
| --- | --- |
| Participants | Setting: outpatient, participants aged 75 and over interviewed at a health center  Source: GeMS Study, Finland  N = 449 subjects cognitively intact at baseline who completed the 3- year follow-up  Users of sedative drugs were older, more likely to be female, live alone, have depressive symptoms and use antidepressant and antipsychotic drugs than non-users |
| Assessments | Exposure to benzodiazepine (BZD) was defined as use over the past 2 weeks: BZD user: regular or as-needed use; non-user no use between baseline and follow-up  Cognitive test at baseline and at follow-up: Mini Mental State Examination (MMSE) |
| Outcomes | Cognitive decline assessed by change in MMSE scores |
| Notes | GeMS: Good Care of the Elderly study |

| ***Critical Appraisal*** | **Yes** | **No** | **Unclear** | **Not applicable** |
| --- | --- | --- | --- | --- |
| 1. Were the two groups similar and recruited from the same population? |  | x |  |  |
| 2. Were the exposures measured similarly to assign people to both exposed and unexposed groups? | x |  |  |  |
| 3. Was the exposure measured in a valid and reliable way? |  | x |  |  |
| 4. Were confounding factors identified? | x |  |  |  |
| 5. Were strategies to deal with confounding factors stated? | x |  |  |  |
| 6. Were the groups/participants free of the outcome at the start of the study (or at the moment of exposure)? | x |  |  |  |
| 7. Were the outcomes measured in a valid and reliable way? |  | x |  |  |
| 8. Was the follow up time reported and sufficient to be long enough for outcomes to occur? |  | x |  |  |
| 9. Was follow up complete, and if not, were the reasons to loss to follow up described and explored? | x |  |  |  |
| 10. Were strategies to address incomplete follow up utilized? |  | x |  |  |
| 11. Was appropriate statistical analysis used? | x |  |  |  |

**Gallacher 2012 [26]**

| Methods | Prospective cohort study |
| --- | --- |
| Participants | Setting: outpatient, participants aged 45-64 years interviewed at home  Source: Caerphilly Study, Wales  N = 1134 men seen on ﬁve occasions over the 22-year follow-up  9.1% reported taking benzodiazepines (BZDs) regularly at one or more phases lasting 4 to 5 years each  BZD users were more likely to be psychologically distressed, have higher levels of trait anxiety and worse cognitive function performance |
| Assessments | Exposure to BZDs was defined as: (1) never use; (2) use at only one examination (referred to as ‘4 years or less’) and (3) use at two or more examinations (referred to as ‘>4 years’)  Cognitive tests at baseline and at follow-ups: AH4 intelligence test, National Adult Reading Test, Four Choice Reaction Time Task, Mini Mental State Examination and Cambridge Cognitive Examination |
| Outcomes | Cognitive decline assessed by change in cognitive scores. Dementia |
| Notes | The study population consisted of men only |

| ***Critical Appraisal*** | **Yes** | **No** | **Unclear** | **Not applicable** |
| --- | --- | --- | --- | --- |
| 1. Were the two groups similar and recruited from the same population? |  | x |  |  |
| 2. Were the exposures measured similarly to assign people to both exposed and unexposed groups? | x |  |  |  |
| 3. Was the exposure measured in a valid and reliable way? |  | x |  |  |
| 4. Were confounding factors identified? | x |  |  |  |
| 5. Were strategies to deal with confounding factors stated? | x |  |  |  |
| 6. Were the groups/participants free of the outcome at the start of the study (or at the moment of exposure)? |  | x |  |  |
| 7. Were the outcomes measured in a valid and reliable way? | x |  |  |  |
| 8. Was the follow up time reported and sufficient to be long enough for outcomes to occur? | x |  |  |  |
| 9. Was follow up complete, and if not, were the reasons to loss to follow up described and explored? | x |  |  |  |
| 10. Were strategies to address incomplete follow up utilized? |  | x |  |  |
| 11. Was appropriate statistical analysis used? | x |  |  |  |

**Mura 2013 [7]**

| Methods | Prospective cohort study, population-based |
| --- | --- |
| Participants | Setting: outpatient, participants aged 65 and over interviewed in a medical center or at home  Source: Three City Study, France  N = 5195 subjects followed up at 2, 4 and 7 years  The mean age was 73.4 years, 59.9% were women  Benzodiazepine (BZD) users (18.6%) were more likely to be women, older, and have depressive and cardiovascular symptoms. They were less likely to have higher educational level and occupational status, smoke or practice physical activity  BZD users presented significantly lower cognitive performances at baseline |
| Assessments | Exposure to BZDs was defined as use in the preceding month more than once a week: (1) non-users: no use at baseline and 2-year follow-up; (2) chronic users: use at both baseline and 2-year follow-up. Exclusion criteria: discontinuation or new prescription at 4 and/or 7-year follow-up  Cognitive tests at baseline and at follow-ups: Mini Mental State Examination, Benton Visual Retention Test, Isaacs Set Test, Trail Making Test, forms A and B |
| Outcomes | Cognitive decline assessed by change in cognitive scores. Cognitive performance. |
| Notes |  |

| ***Critical Appraisal*** | **Yes** | **No** | **Unclear** | **Not applicable** |
| --- | --- | --- | --- | --- |
| 1. Were the two groups similar and recruited from the same population? |  | x |  |  |
| 2. Were the exposures measured similarly to assign people to both exposed and unexposed groups? | x |  |  |  |
| 3. Was the exposure measured in a valid and reliable way? |  | x |  |  |
| 4. Were confounding factors identified? | x |  |  |  |
| 5. Were strategies to deal with confounding factors stated? | x |  |  |  |
| 6. Were the groups/participants free of the outcome at the start of the study (or at the moment of exposure)? |  | x |  |  |
| 7. Were the outcomes measured in a valid and reliable way? | x |  |  |  |
| 8. Was the follow up time reported and sufficient to be long enough for outcomes to occur? | x |  |  |  |
| 9. Was follow up complete, and if not, were the reasons to loss to follow up described and explored? | x |  |  |  |
| 10. Were strategies to address incomplete follow up utilized? |  | x |  |  |
| 11. Was appropriate statistical analysis used? | x |  |  |  |

**Gray 2016 [27]**

| Methods | Prospective cohort study, population-based |
| --- | --- |
| Participants | Setting: outpatient, participants aged 65 and over  Source: Adult Changes in Thought Study, USA  N = 3993 subjects, at least 10 years as a member of the integrated healthcare delivery system, assessed every two years, mean follow-up of 7.3 years  Median age was 74.4 years, 59.6% women, and 66.4% had some college education.  30% had used benzodiazepine (BZD) in the 10 years before study entry, but only 3% had used it in the last six months  Heavier BZD users were more likely to be women and have more depressive symptoms |
| Assessments | Participants were classified as BZD users and non-users. A cumulative total standardized daily dose (TSDD) over the 10-year exposure window was defined for each participant and recalculated at each time point during follow-up. BZD users divided into 3 groups: 1-30 TSDDs, 31-120 TSDDs, or ≥121 TSDDs.  Cognitive test at baseline and at follow-ups: Cognitive Abilities Screening Instrument (CASI) |
| Outcomes | Cognitive decline assessed by change in cognitive scores. Dementia. |
| Notes | The median level of BZD use within the highest BZD use group corresponded to long-term use (slightly over a year of daily use) |

| ***Critical Appraisal*** | **Yes** | **No** | **Unclear** | **Not applicable** |
| --- | --- | --- | --- | --- |
| 1. Were the two groups similar and recruited from the same population? |  | x |  |  |
| 2. Were the exposures measured similarly to assign people to both exposed and unexposed groups? | x |  |  |  |
| 3. Was the exposure measured in a valid and reliable way? |  | x |  |  |
| 4. Were confounding factors identified? | x |  |  |  |
| 5. Were strategies to deal with confounding factors stated? | x |  |  |  |
| 6. Were the groups/participants free of the outcome at the start of the study (or at the moment of exposure)? | x |  |  |  |
| 7. Were the outcomes measured in a valid and reliable way? |  | x |  |  |
| 8. Was the follow up time reported and sufficient to be long enough for outcomes to occur? | x |  |  |  |
| 9. Was follow up complete, and if not, were the reasons to loss to follow up described and explored? | x |  |  |  |
| 10. Were strategies to address incomplete follow up utilized? |  | x |  |  |
| 11. Was appropriate statistical analysis used? | x |  |  |  |

**Chung 2016 [28]**

| Methods | Prospective case-control study |
| --- | --- |
| Participants | Setting: outpatient, 30 nondemented elderly interviewed at Alzheimer’s disease centers  Source: Alzheimer’s Disease Neuroimaging Initiative databases, USA and Canada  Continuous benzodiazepine (BZD) users (n = 15) and matched controls (n = 15) were followed up for 2 years  Mean age 75 years, 73.3% women  Mean duration of BZD use 6.4 years |
| Assessments | Exposure to BZDs defined as: continuous BZD users who continued to use BZDs from baseline to the follow-up endpoint; controls who never used BZDs  Cognitive tests at baseline and at follow-up: Montreal Cognitive Assessment and Auditory Verbal Learning Test |
| Outcomes | Cognitive decline assessed by change in cognitive scores. Levels of cortical β-amyloid |
| Notes |  |

| ***Critical Appraisal*** | **Yes** | **No** | **Unclear** | **Not applicable** |
| --- | --- | --- | --- | --- |
| 1. Were the two groups similar and recruited from the same population? | x |  |  |  |
| 2. Were the exposures measured similarly to assign people to both exposed and unexposed groups? | x |  |  |  |
| 3. Was the exposure measured in a valid and reliable way? |  | x |  |  |
| 4. Were confounding factors identified? | x |  |  |  |
| 5. Were strategies to deal with confounding factors stated? | x |  |  |  |
| 6. Were the groups/participants free of the outcome at the start of the study (or at the moment of exposure)? | x |  |  |  |
| 7. Were the outcomes measured in a valid and reliable way? | x |  |  |  |
| 8. Was the follow up time reported and sufficient to be long enough for outcomes to occur? |  | x |  |  |
| 9. Was follow up complete, and if not, were the reasons to loss to follow up described and explored? | x |  |  |  |
| 10. Were strategies to address incomplete follow up utilized? |  |  |  | x |
| 11. Was appropriate statistical analysis used? | x |  |  |  |

**Zhang 2016 [29]**

| Methods | Prospective cohort study, population-based |
| --- | --- |
| Participants | Setting: outpatient, elderly with normal cognition interviewed at Alzheimer’s disease centers  Source: National Alzheimer’s Disease Coordinating Center’s Uniform Data Set, USA  N = 5423 participants with normal cognition at baseline, mean age 73 years, minimum of 3-year follow-up (mean 4.8 years)  7.5% reported use of a benzodiazepine (BZD) at least once  BZD users were more likely to be white, report alcohol and tobacco use, and have more depressive symptoms |
| Assessments | Exposure to BZDs defined as: no use; any-use (reported BZD use at a minimum of 1 visit); and always-use (reported BZD use at all visits).  Cognitive tests at baseline and at follow-ups: Mini Mental State Examination and Clinical Dementia Rating Sum of Boxes |
| Outcomes | Cognitive decline assessed by change in cognitive scores |
| Notes |  |

| ***Critical Appraisal*** | **Yes** | **No** | **Unclear** | **Not applicable** |
| --- | --- | --- | --- | --- |
| 1. Were the two groups similar and recruited from the same population? |  | x |  |  |
| 2. Were the exposures measured similarly to assign people to both exposed and unexposed groups? | x |  |  |  |
| 3. Was the exposure measured in a valid and reliable way? |  | x |  |  |
| 4. Were confounding factors identified? | x |  |  |  |
| 5. Were strategies to deal with confounding factors stated? | x |  |  |  |
| 6. Were the groups/participants free of the outcome at the start of the study (or at the moment of exposure)? | x |  |  |  |
| 7. Were the outcomes measured in a valid and reliable way? |  | x |  |  |
| 8. Was the follow up time reported and sufficient to be long enough for outcomes to occur? |  | x |  |  |
| 9. Was follow up complete, and if not, were the reasons to loss to follow up described and explored? | x |  |  |  |
| 10. Were strategies to address incomplete follow up utilized? |  |  | x |  |
| 11. Was appropriate statistical analysis used? | x |  |  |  |

**Appendix S5. Characteristics of excluded studies**

| Study | Rationale for exclusion |
| --- | --- |
| Hendler 1980 [32] | Cross-sectional conducted in a clinical sample study assessing cognitive impairment associated with benzodiazepine use rather than cognitive decline |
| Lucki  1986 [33] | Case-control study conducted in a clinical sample assessing cognitive impairment associated with benzodiazepine use rather than cognitive decline |
| Gollombock 1988 [34] | Control group of subjects who had never taken benzodiazepines or who had taken benzodiazepines in the past for less than one year |
| Salzman 1992 [35] | The study addressed the effects of withdrawing from benzodiazepine on cognitive function |
| Tata  1994 [36] | Case-control study conducted in a clinical sample assessing (1) cognitive impairment associated with benzodiazepine use rather than cognitive decline, and (2) the effects of withdrawing from benzodiazepine. Cognitive function assessed pre- and post-withdrawal |
| Gorenstein 1995 [37] | Case-control study conducted in a clinical sample assessing (1) cognitive impairment associated with benzodiazepine use rather than cognitive decline, and (2) the effects of withdrawing from benzodiazepine. Cognitive function assessed pre- and post-withdrawal |
| Foy  1995 [38] | Effects of reported benzodiazepine use in the last month on development of cognitive impairment during hospital stay; subjects had normal cognitive function on admission |
| Berg  1996 [39] | The study addressed the combined effect of multiple medications; specific benzodiazepine effects not assessed |
| Fastbom 1998 [40] | Association between benzodiazepine use and dementia |
| Sumner 1998 [41] | Literature review |
| Rickels  1999 [42] | The study addressed the effects of withdrawing from benzodiazepine on cognitive function |
| Vignola 2000 [43] | Case-control study conducted in a clinical sample assessing cognitive impairment associated with benzodiazepine use rather than cognitive decline |
| Gagné  2000 [44] | French language |
| Lagnaoui 2002 [45] | Association between benzodiazepine use and dementia |
| Wadsworth 2003 [46] | Postal questionnaire study of the association of the use of sleeping tablets and cognitive failure; no cognitive tests were administered and no information about the speciﬁc medication used was available |
| Curran  2003 [47] | The study addressed the effects of withdrawing from benzodiazepine on cognitive function |
| McAndrews 2003 [48] | Case-control study conducted in a clinical sample assessing (1) cognitive impairment associated with benzodiazepine use rather than cognitive decline, and (2) the effects of withdrawing from benzodiazepine. Cognitive function assessed pre- and post-withdrawal |
| Nyström 2005 [49] | Not a study of the specific effects of benzodiazepine use on cognition; a questionnaire was designed to record the patient’s subjective assessments of the effects of long-term benzodiazepine use |
| Barker  2005 [50] | The study addressed the effects of withdrawing from benzodiazepine on cognitive function |
| Puustinen 2007 [51] | Cross-sectional study assessing cognitive impairment associated with benzodiazepine use rather than cognitive decline |
| Bicca  2008 [52] | Portuguese language |
| Wright  2009 [53] | Longitudinal study of the effects of combined use of multiple central nervous system medications on cognition; specific benzodiazepine effects not assessed |
| Lagnaoui 2009 [54] | Association between benzodiazepine use and dementia |
| Tsunoda 2010 [55] | The study addressed the effects of withdrawing from benzodiazepine on cognitive function |
| Deckersbach 2011 [14] | Case-control study conducted in a clinical sample assessing cognitive impairment associated with benzodiazepine use rather than cognitive decline |
| Gnjidic  2012 [56] | The study evaluates the association between Drug Burden Index, a risk assessment tool that measures anticholinergic and sedative medication exposure and cognitive performance, and cognitive impairment; specific benzodiazepine effects not assessed |
| Hoiseth 2013 [57] | Lack of information on the duration of benzodiazepine use. Therefore, subjects may not be long-term users. |
| Farrell  2014 [58] | Case report |
| Tveito  2014 [59] | The study addressed the effects of withdrawing from benzodiazepine on cognitive function |
| Bourgeois 2015 [60] | Group of chronic benzodiazepine users included use for at least 3 months; it meets exclusion criterion (use less than 6 months) |
| Helmes  2015 [61] | Cross-sectional study assessing cognitive impairment associated with benzodiazepine use rather than cognitive decline |
| Federico 2017 [62] | Case-control study conducted in a clinical sample assessing cognitive impairment associated with benzodiazepine use rather than cognitive decline |
